# Biochemical Traits, ^1^H NMR Profile and Residual DNA Content of ‘Asprinio’, White Wine from Campania Region (Southern Italy)

**DOI:** 10.3390/foods11152322

**Published:** 2022-08-03

**Authors:** Nicola Landi, Monica Scognamiglio, Pasqualina Woodrow, Loredana F. Ciarmiello, Sara Ragucci, Angela Clemente, Hafiza Z. F. Hussain, Antonio Fiorentino, Antimo Di Maro

**Affiliations:** Department of Environmental, Biological and Pharmaceutical Sciences and Technologies (DiSTABiF), University of Campania ‘Luigi Vanvitelli’, Via Vivaldi 43, 81100 Caserta, Italy; nicola.landi@unicampania.it (N.L.); monica.scognamiglio@unicampania.it (M.S.); pasqualina.woodrow@unicampania.it (P.W.); lorymail80@libero.it (L.F.C.); sara.ragucci@unicampania.it (S.R.); angela.clemente@unicampania.it (A.C.); hafizazumrafatima.hussain@unicampania.it (H.Z.F.H.); antonio.fiorentino@unicampania.it (A.F.)

**Keywords:** ‘Asprinio’, free amino acids, geographical origin, ^1^H NMR, *Vitis vinifera* L., wine

## Abstract

‘Asprinio’ is a white dry wine characteristic for its acidity and aromatic flavour, known as emerging DOP wine in Southern Italy. Nevertheless, little information is available on the metabolomic profile of this wine. Thus, in this paper we evaluated the colourimetric parameters, ^1^H NMR profiles and free amino acids content of ‘Asprinio’ wines, bottled by two different wineries (hereafter ‘Asprinio_A’ and ‘Asprinio_B’) collected in 2019 and 2020, using ‘Greco di Tufo’ for comparison. The colourimetric parameters are similar for both ‘Asprinio’ wines and differ from ‘Greco di Tufo’ wines. On the other hand, both ^1^H NMR and free amino acid content profiles show different chemometric profiles among the three wines analysed, although the profiles are similar for both vintages. Moreover, the multivariate analyses carried out highlight differences between ‘Asprinio_A’ and ‘Asprinio_B’, which exbibit also different residual yeast and plant DNA. Overall, considering that the two-manufacturing wineries use 100% ‘Asprinio’ grape, the difference retrieved between the two ‘Asprinio’ wines could be explained by the different grapevine training systems: ‘*vite maritata*’ (training system inherited from Etruscans) for ‘Asprinio_A’ and ‘*guyot*’ for ‘Asprinio_B’.

## 1. Introduction

Wine is an alcoholic drink obtained by *Vitis vinifera* L. fermentation of total, partial juice or solid grape [1]. In this water-based mixture, various compounds are found in the solution, while others are present in the colloidal state, all contributing to the different features of white and red wines [2].

Wine is principally constituted by water (~85%) and ethanol (~range 11–14%), the latter produced by yeasts during the fermentation process. In addition, other compounds (~3%) such as sugars (glucose, fructose and several non-fermentable pentoses), glycerol, organic acids (tartaric acid, malic acid, lactic acid, succinic and citric acid), phenolic and volatile compounds (terpenoids, phenols, alcohols, esters, aldehydes, ketones and lactones) as well as inorganic ions are present in minor amounts [3,4]. According to the period of their formation, most of them are classified as: (i) primary, in the grape; (ii) secondary, derived from alcoholic or malolactic fermentation from yeast or bacteria, respectively; and (iii) tertiary, during storage (e.g., Oak lactones, trichloroanisole and C_13_-norisoprenoids family) [4,5].

Flavour (or wine’s aroma) and quality of white and red wines are definitely related to the content of these compounds, depending on multiple parameters such as grape variety, ripeness degree at the time of harvesting as well as pedoclimatic characteristics (e.g., location, soil, temperature and agronomic practices) [6,7,8]. In addition, they are affected by the diversity of local yeast cultures and other microorganisms responsible for alcoholic fermentation. Indeed, *Saccharomyces cerevisiae* and non-*Saccharomyces* yeast species/strains coexist and interact during the fermentation course influencing the analytical profile and wine’s aroma [9]. On the other hand, wine production is not only linked to economic gains but also to local history and traditions, for which wine contributes to promoting the territories of origin [10]. Indeed, several wines have become the history of people producing them, as well as the essence of their traditions [11].

An example of symbiosis between wine properties and territories of production is well represented by Italy, in which microclimates and geologically diverse soils as well as a large number of native grape varieties allowed the production of many wines known all over the world. Indeed, many Italian wine appellations, such as Barolo and Barbaresco (Piedmont), Valtellina superiore and Franciacorta (Lombardy), Amarone della Valpolicella (Veneto), Brunello di Montalcino (Tuscany) and other notable wines from the Centre and Southern Italy such as Verdicchio (Marche), Sagrantino (Umbria), Taurasi (Campania), Primitivo and Nero d’Avola (Puglia) are getting attention in recent years [12].

Nowadays, also several appellations produced in the Campania region (Southern Italy) have gradually attracted the favour of consumers considering the local grape varieties and peculiar pedoclimatic conditions [12]. In particular, the most popular appellations among international market and specialists are: Aglianico del Taburno (red), Greco di Tufo (white), Fiano di Avellino (white), Falanghina del Sannio (white), Asprinio d’Aversa (white; commonly called ‘Asprinio’) and many others [13]. Among them, ‘Asprinio’ wine is obtained from the native grape variety, deriving from the domestication of wild vines thousands of years ago [14]. ‘Asprinio’ (from the Latin term ‘asper’) is a very light (~11% alcohol level) high-acid, dry, fresh wine and its flavour is significantly aromatic [15]. ‘Asprinio’ is also a versatile white wine considering the culinary heritage of the Campania region. It goes well with seafood, especially seasoned with lemon or vinegar, and it is perfect in combination with the local buffalo mozzarella, sweet tomatoes and aromatic basil. ‘Asprinio’ wine production has mainly spread in 19 communes, known as ‘agro-aversano’ territories (located in northern Campania, Caserta province), while only 3 are located in Napoli province. The traditional training system provides that vines are trained to poles, and may grow 10 m in the air. Indeed, the vines for ‘Asprinio’ grapes production are known as ‘piantata’ where the foliage winds up between the trees, often tall poplars, with the help of steel wires. This particular training system, inherited from the Etruscans is known generally as ‘*vite maritata*’ or ‘alberata d’Asprinio’, and is today preserved as a cultural heritage [16]. This technique gives the grapes maximum sunlight, providing shade and protection to the other crops, which are planted between the widely spaced vine rows. Recently, the study of ‘Asprinio’ grape variety origin is receiving new impetus considering as reported by Spada and co-authors. They highlighted the quality of this vine/wine, proposing similar traits with Greco by morphological analysis [17,18]. Greco is a white grape variety mainly grown in the Campania region, the wines obtained can vary from fresh and herbal to full-bodied with hints of stone fruit. Greco vine is cultivated in several territories of this region and is often associated with ‘Greco di Tufo’ (Tufo, province of Avellino) [18]. Moreover, other researchers consider that the two vines are identical, although wine’s aromatic peculiarities occur due to the differences in both production territories and training systems [19,20], however, other researchers disagree [15,21]. Finally, considering the particular pedoclimatic characteristics of the cultivation area and the production method, it is decided that the ‘Asprinio’ is a Controlled Designation of Origin since 1993 (D.O.C. in Italy) [22].

In this framework, the aim of this study was to characterize for the first time ‘Asprinio’ wine considering its biochemical traits (colourimetric parameters, free amino acids amount, antioxidant capacity, total phenol, flavonoids and tannin content.), Nuclear Magnetic Resonance (NMR) profile and the residual DNA content. For this purpose, ‘Asprinio’ wine bottled by two different wineries (hereafter ‘Asprinio_A’ and ‘Asprinio_B’) was selected and then analysed considering two grape harvests (2019 and 2020). In particular, the two wineries have been chosen considering the different ‘Asprinio’ grapevine training systems. Indeed, ‘Asprinio_A’ grapes are cultivated with the traditional ‘*vite maritata*’ training system, while for ‘Asprinio_B’ grape is used the ‘*guyot*’ training system (recently used for the ‘Asprinio’ vine), see Figure S1a,b, respectively. As a reference, the same analyses were performed on ‘Greco di Tufo’ wine bottled and marketed by a well-known winery, used for comparison.

## 2. Materials and Methods

### 2.1. Chemicals and Reagents

All chemicals were obtained from Sigma-Aldrich (St. Louis, MO, USA). Chemicals and buffers for the automated amino acid analysis were provided by ERRECCI (Opera, Milano, Italy).

### 2.2. Wine Samples

Two commercially available ‘Asprinio’ wines obtained from two different grape harvests (2019 and 2020) were selected and analysed. The bottles of wine were produced by two different wineries (called ‘Asprinio_A’ and ‘Asprinio_B’) located in ‘agro-aversano’ territories (province of Caserta, Campania, Italy). All bottles (3 per year for each winery; total of 12 bottles) were purchased in December, stored at 10 °C in the dark and analysed shortly after opening. As a reference, we used ‘Greco di Tufo’ wine obtained from two grape harvests (2019 and 2020) produced by a well-known Avellino winery (Campania, Italy). The description of these wine samples is reported in Table S1.

### 2.3. pH Determination

A Crison GLP21 pH meter was used to determine the pH value. Results obtained are the average of three determinations (Regolamento CEE n.2676/90 della Commissione del 17 settembre 1990, Gazzetta ufficiale n. L 272 del 03/10/1990).

### 2.4. Spectrophotometric Assays and Antioxidant Capacity

All spectrophotometric measurements were performed in UV-VIS Agilent Cary 100 Spectrophotometer (Agilent Technologies, Santa Clara, CA, USA).

#### 2.4.1. Total Phenolic Content (TPC)

TPC of the three different wines was determined according to the Folin-Ciocalteu procedure [23]. TPC value of samples was expressed as mg of gallic acid equivalents (GAE) per L of wine using a calibration curve in the concentration range of 2.5–100 mg/L. Briefly, 200 μL of Folin-Ciocalteu reagent (10%; *v*/*v*) were added to 100 μL of wine sample previously diluted (2-fold dilution for each wine). After 5 min incubation, 800 μL of sodium carbonate (7.5%; *w*/*v*) were added. The whole mixture was then incubated at room temperature in the dark for 2 h. The mixture was then centrifuged at 18,000× *g* for 4 min and the supernatant was transferred into the cuvette to measure the absorbance at 725 nm using methanol as blank.

#### 2.4.2. Total Tannins Content (TTC)

TTC was determined by following a protocol described previously [24]. For each wine, two samples containing 200 μL of 10-fold-diluted wines, 300 μL of 37% HCl, and 100 μL of distilled water were prepared. The first sample was incubated at 100 °C for 30 min, whereas the second sample was left at room temperature with the addition of 50 μL ethanol. The absorbance of both samples was measured at 470, 520, and 570 nm. The differences in absorbance between the two samples at a given wavelength were calculated and represented as ΔA_470_, ΔA_520_, and ΔA_570_. ΔA_470_ and ΔA_570_ were then expressed in terms of ΔA_520_ using the following formula: ΔA_520_ = 1.1 × ΔA_470_ and ΔA_520_ = 1.54 × ΔA_470_. The lowest ΔA_520_ value was chosen to estimate TTC as g/L of wine using the following equation: TTC = 15.7 × lowest ΔA_520_.

#### 2.4.3. Total Flavonoid Content (TFC)

TFC was determined using the procedure described by Di Stefano et al., 1989 [25]. Briefly, ‘Greco di Tufo’ wine was diluted at 1:25, while ‘Asprinio_A’ and ‘Asprinio_B’ wines were diluted at 1:20 with 99% ethanol acidified with 0.1% HCl. For each wine sample, the spectrum was registered in the range of 230–700 nm. Subsequently, the spectra obtained were corrected with a graphical method to eliminate the contribution of interfering substances that absorb at the same wavelength. Finally, the TFC value was expressed as mg/L of (+)-catechin using the following equation: TFC = (A_280_ × 82.4 × *d*) where A_280_ was the absorbance at 280 nm corrected with the graphical method; 82.4 is the ratio between the concentration of a (+)-catechin standard solution and its corrected absorbance and *d* indicates the dilution factor.

#### 2.4.4. Colorimetric Parameters

The parameters of wine colour were estimated by measuring the absorbance of wine samples at 420, 520 and 620 nm [23,26,27]. The sum of absorbance at 420 and 520 nm was expressed as colour density (IC), whereas the sum of absorbance at 420 nm, 520 nm, and 620 nm was referred to as colour intensities (IC’). The proportions of red (%R), yellow (%Y), and blue (%B) were determined by using the following formula: %*R* = [(A_520_ × 100)/IC′]; %Y = [(A_420_ × 100)/IC′]; %B = [(A_620_ × 100)/IC′]. Wine colour (WC) was determined by adding 20 μL acetaldehyde to 2 mL wine and measuring its absorbance at 520 nm after 45 min. The wine total colour of pigments (WCP) was determined by adding 900 μL of 0.1 M HCl to 100 μL wine and measuring its absorbance value at 520 nm after 4–5 h. Wine polymeric pigment colour (WPPC) was determined by adding 15 mg sodium bisulphite to 5 mL wine and measuring its absorbance at 520 nm after 1 min.

#### 2.4.5. ORAC Assay

The antioxidant potential of ‘Asprinio_A’, ‘Asprinio_B’ and ‘Greco di Tufo’ wines, was measured by the oxygen radical absorbance capacity (ORAC) assay [28]. The analysis was performed using a 96-well microplate in which 25 μL of each sample was appropriately diluted. The fluorescence decay was recorded by using a Synergy HT multi-mode microplate reader (BioTek, Bad Friedrichshall, Germany). ORAC values were calculated using the Trolox^®^ calibration curve and expressed as Trolox^®^ equivalents (mmol Trolox per L of wine).

### 2.5. NMR Methods

NMR samples were prepared as follows: for each sample, 100 µL of phosphate buffer (90 mM; pH 6.0) in D_2_O (Sigma-Aldrich) containing 0.1% *w*/*v* trimethylsilylpropionic-2,2,3,3-*d*_4_ acid sodium salt (TMSP, Sigma-Aldrich) were added to 900 µL of wine. Samples were vortexed and 600 µL were transferred to NMR tubes for subsequent analysis. Each wine type and each vintage were analysed in triplicate.

NMR spectra were recorded at 25 °C on a Bruker 300 Fourier transform NMR operating at 300.03 MHz for ^1^H and 75.45 MHz for ^13^C. D_2_O was used as an internal lock. No sample rotation was applied. The 1D ^1^H NMR spectra were acquired using a WET 1D sequence to suppress both the water CH_3_ and CH_2_ signals from ethanol.

Free induction decays (FIDs) were Fourier-transformed and the resulting spectra were manually phased, baseline-corrected and calibrated to TMSP at 0.0 ppm, using a ^1^H NMR processor (ACDLABS 12.0, Toronto, ON, Canada).

^1^H NMR spectra were scaled to total intensity, bucketed, and reducing them to integral segments with a width of 0.04 ppm with ACDLABS 12.0 ^1^H NMR processor (ACDLABS). The regions at δ −0.02–0.02, 0.90–1.42, 3.30–3.92, and 4.70–5.00 were excluded from the analysis (by indicating them as dark regions before integration) because residual TMSP, ethanol and water signals.

### 2.6. Free Amino Acid Composition

Free amino acid composition of ‘Asprinio_A’, ‘Asprinio_B’ and ‘Greco di Tufo’ wines were obtained by sampling 1 mL (in triplicate) per bottle. Subsequently, the samples were freeze-dried for amino acid extraction. The freeze-dried powder was subjected first to ethanol precipitation using 80% cold ethanol (1.0 mL), in the presence of *nor*-leucine (200 nmol) as internal standard, dissolved with a Teflon pestle, and centrifuged at 14,000× *g* at 4 °C. The supernatant was lyophilized, treated with 3% sulfosalicylic acid (500 μL) to precipitate any protein fraction still present, and recentrifuged [29,30]. Aliquots of samples (generally 30 μL) were directly analysed on a Biochrom-30 amino acid analyser (Biochrom, Cambridge, UK), equipped with a post-column ninhydrin derivatization system [31].

### 2.7. DNA Wines Analysis

#### 2.7.1. DNA Wines Extraction

1 vol of 2-propanol was added to 100 mL of wine, precipitated 1 week at −20 °C and centrifuged at 13,000× *g* for 30 min at 4 °C. The pellet was dissolved in 1 mL preheated extraction buffer [20 mM EDTA, 10 mM Tris-HCl pH 8.0, 1.4 M NaCl, 1% CTAB, 3% PVP-40 and 1% β-mercaptoethanol added just before use), incubated at 60 °C for 60 min mixing the samples every 10 min. After incubation, 1 vol of phenol:chloroform:isoamyl alcohol (25:24:1) (*v*/*v*/*v*) was added to the sample for the extraction, mixed for 15 min on a rotary shaker and centrifuged at 13,000× *g* for 10 min at 4 °C. The upper layer phase was subjected again to centrifugation at 5000× *g* for 5 min to remove the excess PVP-40 from the samples. The aqueous phase was transferred to another tube containing 0.6 vol of ice-cold 2-propanol, the tubes were mixed gently by inverting several times and the samples were precipitated overnight at −80 °C. After precipitation, the pellet was collected by centrifugation at 13,000× *g* for 20 min at 4 °C and dissolved in 500 μL of 1× TE buffer (10 mM Tris•Cl, 1 mM EDTA, pH 8.0). An equal volume of neutral phenol was added to the sample followed by centrifugation at 13,000× *g* for 15 min at 4 °C. The upper layer phase was subjected again to centrifugation at 13,000× *g* for 15 min at 4 °C. The aqueous phase containing the nucleic acid was precipitated with 2 vol ice-cold 76% ethanol containing 10 mM ammonium acetate for 30 min at −80 °C. After precipitation, DNA was collected by centrifugation at 13,000× *g* for 20 min at 4 °C. DNA pellet was dried at room temperature and resuspended in 50 μL 1× TE buffer. DNA quantification and quality were determined by NanoDrop ND-1000 spectrophotometer (NanoDrop Technologies, Wilmington, DE, USA).

#### 2.7.2. DNA Grapevine and Yeast Quantification

DNA quantification was carried out by quantitative PCR analyses using SYBR Green qPCR Mix (high ROX; GDSBio) by Applied Biosystems™ QuantStudio™ 5 Real-Time PCR System (Thermo Fisher Scientific, Rodano (MI), Italy). The reaction mix (20 µL) consisted of: 2× SYBR Green qPCR Mix, Forward 10 µM for each primer, and DNA template 50 ng. The amplification protocol was: 95 °C initial denaturation step for 3 min, followed by amplification cycles (40×) of denaturing at 95 °C for 10 s, annealing at 60 °C for 10 s and extension at 72 °C for 20 s. Amplification products were visualized on 1.5% (*w*/*v*) agarose gel, using a UV light. Primers used in this study are listed in Table S2.

### 2.8. Statistical Analysis

Each experiment was repeated three times, and the results are reported as mean ± standard deviation. The radar graph was obtained by using Excel 2016 Microsoft (Redmond, WA, USA). In order to identify groups related to the free amino acid profiles, a multivariate cluster analysis was carried out, using the statistical package PAST_4 [32]. Dendrograms were built using clustering with Ward’s method (clusters are joined such that the increase in within-group variance is minimized). Pearson correlation was used for the analysis of the correlation between various parameters and the significance of correlation using the GraphPad Prism 8 software (GraphPad Software Inc., San Diego, CA, USA). The Bonferroni post-test was used to determine significant differences. The test was performed using a *p* < 0.05.

Principal component analysis (PCA) was performed with the SIMCA-P software (version 14.0, Umetrics, Umeå, Sweden) with scaling based on Pareto.

## 3. Results and Discussion

### 3.1. Colorimetric Parameters

The colour of the wine is one of the most important visual characteristics of consumer choices. Furthermore, this parameter mainly depends on the colour of the grape variety. The colourimetric parameters of the three white wines analysed for 2019 and 2020 vintages, determined in terms of wine colour (WC), wine polymeric pigment colour (WPPC) and wine total colour of pigments (WCP) were reported in Table 1. Data showed that the colourimetric parameters of the two different vintages were similar for each wine analysed. On the other hand, considering the average parameters of the three wines for both vintages, WC and WCP values of ‘Asprinio_A’ and ‘Asprinio_B’ wines were 3-fold higher than ‘Greco di Tufo’ wine values, while WPPC values are similar for all wines analysed and did not exceed 0.003 AU. 

Subsequently, wine colour was measured in terms of red, yellow and blue percentages (Table 1). Data obtained showed that in all wines analysed, the most abundant colour percentage corresponded to yellow (~80%), followed by red (~17%) and blue (~3%). In particular, ‘Greco di Tufo’ wine had a higher percentage of yellow colour (~85%) with respect to ‘Asprinio_A’ and ‘Asprinio_B’ wines (both ~77%). In addition, WC values showed a strong positive correlation with total polyphenolic content (*r* = 0.9261; *p* < 0.001, see later) and total tannin content (*r* = 0.9044; *p* < 0.05; see later) values, while there is no correlation with total flavonoids content (*r* = −0.03583; *p* = 0.9463). No significant correlation of WC with total flavonoid content indicates that flavonoids do not contribute to wine colour. Even if it is known that the colour of white wines is influenced by their flavonoids [33]. The positive correlation retrieved between WC and total polyphenolic content was in agreement with previously reported observations [23].

### 3.2. Total Polyphenols, Tannins, Flavonoids Content and Antioxidant Capability

**Figure 1:**
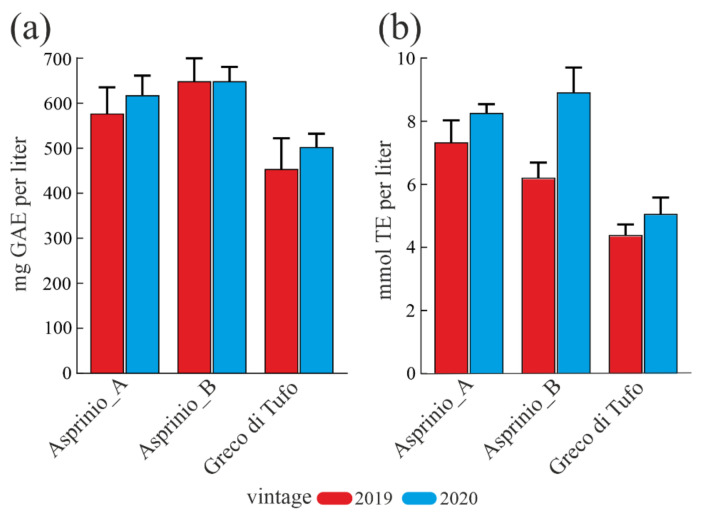
Total phenol content and antioxidant capabilities in ‘Asprinio_A’, ‘Asprinio_B’ and ‘Greco di Tufo’, considering the 2019 and 2020 vintages. (**a**), total phenol content (TPC) expressed as mg of gallic acid equivalents (GAE) per L of each wine ± SD. (**b**) Trolox equivalent (TE) antioxidant activity from ORAC assay expressed as mmol of Trolox equivalents (TE) per L of each wine ± SD. boxed.

Polyphenolic compounds influence the organoleptic characteristics of wines. In light of this, the total polyphenolic content (TPC) of the three white wines analysed for 2019 and 2020 vintages was estimated by using the Folin-Ciocalteu assay and TPC values are reported in Figure 1a. TPC was higher in the 2020 vintage compared to 2019 for both ‘Geco di Tufo’ and ‘Asprinio_A’ wines, while it is similar for ‘Asprinio_B’ wines for both vintages. In particular, the average TPC value of ‘Asprinio_B’ wine was higher than those of ‘Asprinio_A’ and ‘Greco di Tufo’ wines (647.72 mg/L, 596.88 mg/L and 477.45 mg/L, respectively).

Subsequently, the total tannin content (TTC) of the three white wines analysed for 2019 and 2020 vintages was evaluated showing that TTC values for each wine were similar between the two vintages, although TTC values of ‘Asprinio_A’ and ‘Asprinio_B’ wines were higher than ‘Greco di Tufo’ wine (~5-fold and ~3-fold, respectively), Table 1. On the other hand, the total flavonoid content (TFC) of wine samples has an equal average value for the analysed wines (Table 1). In particular, TFC values were higher in the 2020 vintage compared to 2019 for all analysed wines.

Finally, the antioxidant activity of wine varieties was evaluated by using the ORAC assay. The data showed a similar trend in the antioxidant activity for all wines analysed, while a different trend was found comparing the two different vintages, Figure 1b. Indeed, an evident antioxidant activity was observed for the 2020 vintage compared to the 2019 vintage. Furthermore, ‘Asprinio_A’ and ‘Asprinio_B’ wines displayed higher antioxidant power compared to ‘Greco di Tufo’ wine, for both vintages analysed.

The average antioxidant power in ‘Asprinio’ wines was found to be 6.8 and 8.6 mmol TE/L, for 2019 and 2020 vintages, respectively; which is 1.5 and 1.7-fold higher than ‘Greco di Tufo’ wine, for 2019 and 2020 vintages, respectively. However, considering polyphenols content and wine antioxidant power, we confirm a correlation between TPC and antioxidant activity of ‘Asprinio_A’, ‘Asprinio_B’ and ‘Greco di Tufo’ wines due to increment of both parameters.

### 3.3. Metabolic Profiling

**Figure 2:**
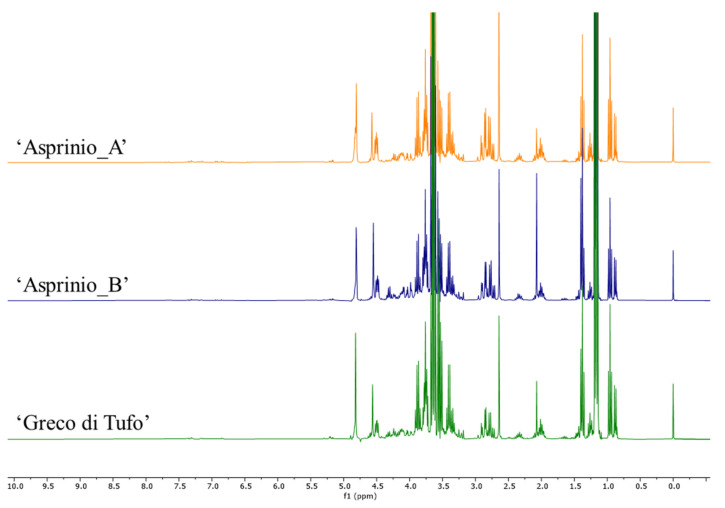
^1^H NMR spectra of 3 representative samples of ‘Asprinio_A’, ‘Asprinio_B’ and ‘Greco di Tufo’.

In order to rapidly and effectively compare the wine samples based on their metabolite content, NMR-based metabolomics was carried out. This approach finds application in agriculture and in food analysis, due to its versatility, high-throughput nature, reproducibility, ease of sample preparation, together with the possibility to obtain reliable structural information even in a mixture [34]. Recent publications reported the use of NMR for quality control studies, authenticity, or geographical characterization of different foodstuff (e.g., olive oil [35], beer [36] and wine [37]). In the case of wine samples, it is possible to perform the analysis with minimum sample preparation. Indeed, the samples were spiked with the deuterated solvent and directly analysed by ^1^H NMR. The metabolites were identified in the ^1^H NMR spectra (Figure 2; Table S3) based on the comparison with data reported in the literature [37,38,39,40].

**Figure 3:**
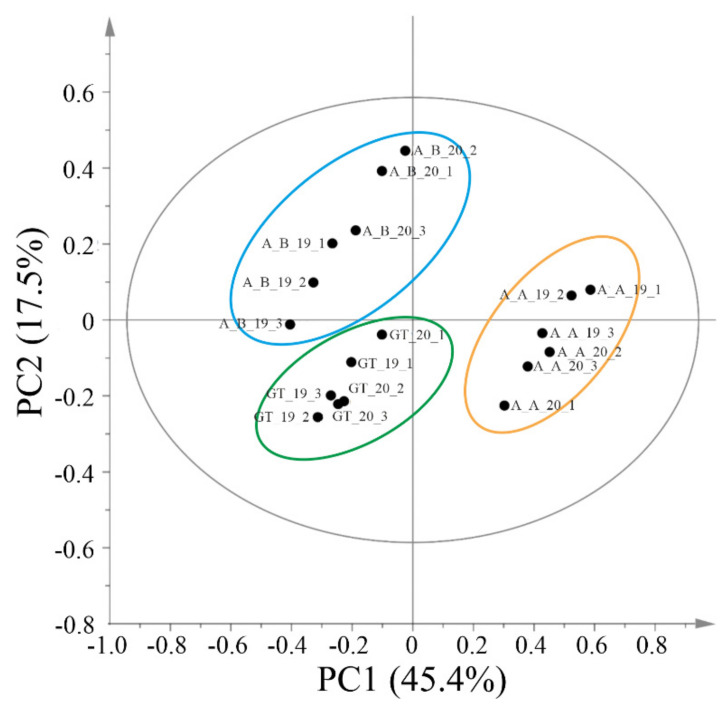
Score scatter plot of PC1 versus PC2 obtained from the PCA of NMR data. The ellipse represents the Hotelling T2 with 95% confidence. A_A = ‘Asprinio_A’; A_B = ‘Asprinio_B’; GT = ‘Greco di Tufo’. The vintage is indicated by the numbers 19 (for 2019) and 20 (for 2020).

Principal component analysis of ^1^H NMR data was carried out and the PCA score plot showed the presence of 3 clusters A_B, GT and A_A. The latter cluster was separated from A_B and GT along PC1, while A_B and GT were separated along PC2 (Figure 3).

Each cluster was composed of samples of a single wine type, independently from the vintage. Therefore, the main differences observed among the analysed samples are attributable to wine rather than to the vintage. The two ‘Asprinio’ wines, ‘Asprinio_A’ and ‘Asprinio_B’ produced by two different wineries, formed two groups at the two different sides of the first principal components.

The main metabolites responsible for the separation along this component were some organic acids; malic (2.70–2.90 ppm and 4.48–4.52 ppm) and citric acids (signals overlapping with those of malic acid at 2.70–2.90 ppm) were positively correlated with PC1. On the other hand, tartaric (δ 4.55, s) and acetic acid (δ 2.08, s) were negatively correlated with this component. In addition, differences were detected also for the amino acids which were then analysed by targeted approaches (see Section 3.4).

**Figure 4:**
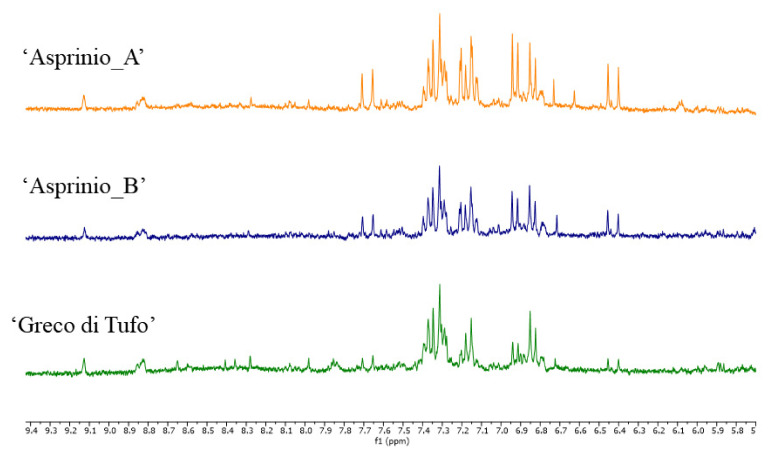
^1^H NMR spectra of 3 representative samples of ‘Asprinio_A’, ‘Asprinio_B’ and ‘Greco di Tufo’, details of the aromatic region.

PCA components did not show specific correlations with buckets in the aromatic region. However, the compounds responsible for these signals were identified. In particular, in the aromatic region of the spectra (Figure 4) caffeic acid was identified by detecting the signal at δ 7.68 (d, J = 16.0), 7.21 (d, J = 2.0), 7.14 (dd, J = 8.0 and 2.0), 6.84 (d, J = 8.0), and 6.43 (d, J = 16.0) [38]. The multiplets between 7.2 and 7.4 ppm were likely attributable to 2-phenylethanol, while two doublets at δ 6.93 and 7.19 (J = 7.7) were attributable to tyrosol [39]. Finally, signals belonging to traces of trigonelline were detected in all the wines analysed [41].

Overall, metabolomics analysis showed that the metabolite content of the three analysed wines was very similar, with differences that were mainly due to the relative amounts of certain primary metabolites. The observed separation of the two Asprinio wines suggests that the different grapevine training systems affect the wine chemical composition of this cultivar.

### 3.4. Free Amino Acids Content

The free amino acid content in wine depends on several factors such as different grape varieties, amino acid consumption and release by yeasts during fermentation, as well as protein digestion by endoproteinases released after yeasts autolysis [42]. However, considering that free amino acid content represents ~40% of the total nitrogen in wines, it influences wine flavour [43].

In this framework, a comparison of ‘Asprinio_A’, ‘Asprinio_B’ and ‘Greco di Tufo’ free amino acid content was carried out by using cation exchange liquid chromatography and post-column ninhydrin derivatization.

The analyses show quantitative differences for the three wine samples (Table 2), although there are no qualitative differences among all protein amino acids, except for cysteine and tryptophan, which are not detected. Moreover, no qualitative differences among all non-protein amino acids such as phosphoserine (Phser), taurine (Taur), β-ala (β-alanine), β-aminoisobutyric acid (Baiba), homocysteine (homocys), gamma-aminobutyric acid (Gaba), ethanolamine (Ethan) and ornithine (Orn) were retrieved. Total free amino acid content for ‘Asprinio_A’, ‘Asprinio_B’ and ‘Greco di Tufo’ wines was 179.48, 97.28, and 198.34 mg/L, respectively, for the 2019 vintage, while 89.72, 49.67, and 134.46 mg/L, respectively, for the 2020 vintage. Furthermore, the proline (Pro) concentration for the two vintages, is reported separately (Table 2) considering that it is predominantly released by yeasts [42,44]. However, the average proline concentration (~169 mg/L) for the 2019 vintage is lower than the 2020 vintage (~247 mg/L).

The free amino acid content of ‘Asprinio_A’ wine obtained by analysing both 2019 and 2020 vintages has no statistical differences, except for glutamine (Gln), lysine (Lys) and arginine (Arg), despite the different amino acids concentration. Vice versa, the free amino acid content of ‘Asprinio_B’ wine obtained by analysing both 2019 and 2020 vintages, exhibits a different profile for several amino acids (~40%) showing statistical differences [i.e., aspartic acid (Asp), serine (Ser), glutamic acid (Glu), Gln, alanine (Ala), leucine (Leu), Lys, β-ala, homocys and Gaba]. On the other hand, the free amino acid content of ‘Greco di Tufo’ wine obtained by analysing both 2019 and 2020 vintages shows slight statistical differences only for Gln and Lys (*p* < 0.05).

**Figure 5:**
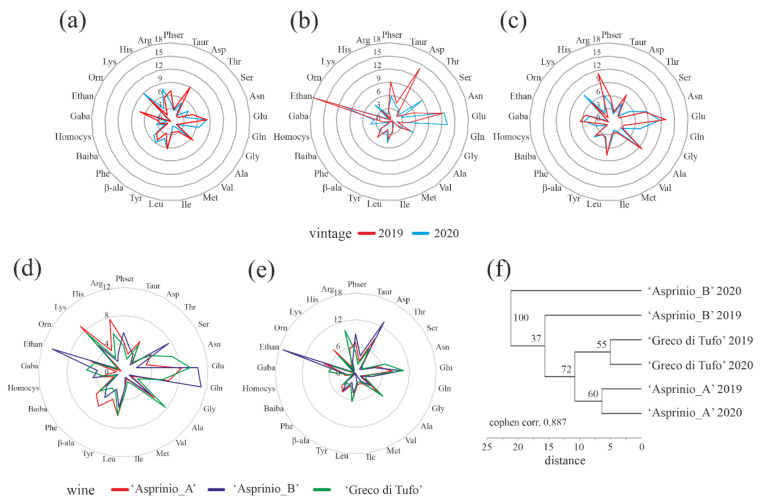
In (**a**–**c**), radar graph representing the free amino acid profile changes (percentage) in ‘Asprinio_A’, ‘Asprinio_B’ and ‘Greco di Tufo’, respectively, analysed in 2019 and 2020 vintages. In (**d**,**e**), radar graph representing the free amino acid profile changes of ‘Asprinio_A’, ‘Asprinio_B’ and ‘Greco di Tufo’, respectively, in 2019 or 2020 vintages. In (**f**), dendrogram corresponding to the cluster analysis of free amino acid profiles in ‘Asprinio_A’, ‘Asprinio_B’ and ‘Greco di Tufo’ from 2019 and 2020 vintages. Numbers on the dendrogram are bootstrap values (% of 1000 replicates).

In this framework, in order to obtain possible relation among free amino acid profiles of ‘Asprinio_A’, ‘Asprinio_B’ and ‘Greco di Tufo’ wines from 2019 and 2020 vintages, the percentages of free amino acids, were subjected to comparative analyses, except for Pro content, as displayed by radar graphs in Figure 5a–c, respectively. This graphical representation highlights similar free amino acid profiles for each wine per vintage analysed, while ‘Asprinio_A’, ‘Asprinio_B’ and ‘Greco di Tufo’ free amino acid profiles exhibited differences between the two vintages by superimposition, Figure 5d,e. Indeed, the dendrogram obtained by using relative cluster analysis suggested three groups, each constituted by two different vintages profiles of ‘Asprinio_A’, ‘Asprinio_B’ and ‘Greco di Tufo’ wines, Figure 5f, in which ‘Asprinio_A’ free amino acid profile significantly differed from ‘Asprinio_B’ and is related to ‘Greco di Tufo’.

### 3.5. Quantification of Endogenous Genes from Grapevine and Saccharomyces cerevisiae

The genuineness of the wine can be verified on the bases of DNA analysis because genetic characterization provides information on a specific variety or presence of foreign species at lower concentrations. In fact, the variety used raises the price of wine on the market [45]. Since DNA is not influenced by the environment and cultural practices, it provides the best tool for unmasking fraud [46]. Scientific literature reported studies carried out under controlled conditions (wines obtained through micro vinification, by artificially mixing a known amount of specific varieties) and few papers reported real cases [46,47]. Since wine genotyping of the varietal composition identification is important for product traceability and certification mechanism, in the present study, DNA was extracted from three different varieties of wines: ‘Asprinio_A’, ‘Asprinio_B’ and ‘Greco di Tufo’. Since DNA extracted from wine samples contained both vegetable and yeast DNA, the quantification was determined by real-time PCR assay. For yeast identification, *S. cerevisiae* ribosomal Protein 3 gene (*ScRPS3*) (GenBank AN: U34347) primers were used [48]. For plant identification, a primer pair developed on the *V. vinifera* nine-cis-epoxycarotenoid dioxygenase2 gene (*VvNCED2*) deposited in the Gene bank under AN: AY337614, was used. A third primer pair combination was designed on the Internal transcribed Spacer region of nuclear ribosomal DNA (ITS1) universal for yeast and plants [49]. All qPCR analyses were performed as triplicates on three independent replicates.

**Figure 6:**
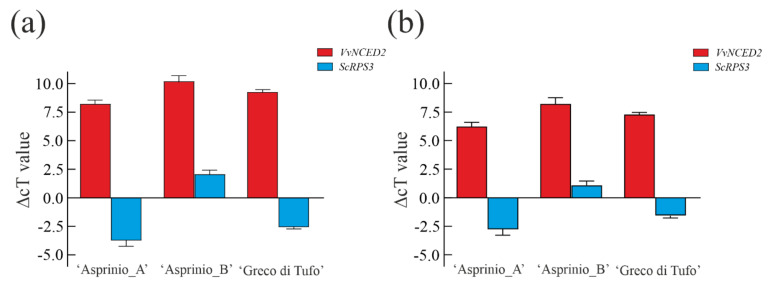
In (**a**,**b**), relative quantification of grapevine and yeast DNA in ‘Asprinio_A’, ‘Asprinio_B’ and ‘Greco di Tufo’ samples in 2019 and 2020 vintages, respectively. Data are means of relative quantification measurements based on the ∆Ct Method ± SD; *n* = 3 biological replicates for each sample.

As shown in Figure 6, the sizes of real-time products for both vintages, reveal a good amplification signal in all samples, also confirmed by agarose gel electrophoresis analysis (data not shown). As expected, the highest amplification signals were obtained with ITS1 universal primer for both plant and yeast in all samples. Instead, the yeast-specific primer pair combination showed a low signal intensity in all wine samples. qPCR quantification was performed using the specific primers set of the *VvNCED2* and *ScRPS3* genes, as they represent a good candidate for the detection of wine endogenous genes [48]. *VvNCED2* and *ScRPS3* were normalized using the ITS1 gene as a reference for plants and yeast. Yeast and grapevine DNA quantification was determined by subtracting Ct value of *VvNCED2* and *ScRPS3* genes from Ct values of the ITS1 reference gene (Figure 6).

These results highlight a high Ct value for the *VvNCED* gene and a lower Ct value for the *ScRPS3* gene, for all samples, indicative of a different amount of grapevine and yeast DNA in the wine samples. In particular, considering the three wines analysed, ‘Asprinio_A’ samples showed the lowest yeast Ct, whereas ‘Asprinio_B’ showed the highest grapevine Ct value, for both vintages. This result can be correlated to the different amino acid content present in the two wines because *Saccharomyces* and non-*Saccharomyces* yeasts have a specific amino-acid consumption profile. Thus, differences in DNA contents are most likely due to sources of yeast assimilable nitrogen (YAN) and the nitrogen demand required by the yeast strain. The differences between grapevine or yeast DNA contents are most likely due to the different processes involved in winemaking. Indeed, it is known that biotic and abiotic mechanisms affect yeast-yeast interactions during wine fermentation, determining, in a natural process, the final dominance of one or few yeast strains [50]. In fact, there are yeast strains capable of consuming the nitrogen and carbon sources more quickly available in grape juice, together with the high tolerance to ethanol, compared to others. Thus, during fermentation processes, the specific amino acid consumption by non-*Saccharomyces* yeasts may justify the presence of different DNA contents of *S. cerevisiae* [51].

## 4. Conclusions

In this study, we reported for the first time the characterization of ‘Asprinio’, white DOP wine from the Campania region, evaluating its biochemical traits, ^1^H NMR profile and residual DNA content. In particular, we analysed ‘Asprinio’ wines bottled by two different wineries, named ‘Asprinio_A’ and ‘Asprinio_B’, produced with two different grapevine training systems, ‘vite maritata’ (also known as ‘alberata d’Asprinio’) and ‘guyot’ method, respectively. Our data revealed quantitative differences among metabolites retrieved from these wines, although the profiles are similar. Therefore, the difference between the two ‘Asprinio’ wines is likely due to the variability in yeasts population (type or quantity), confirmed by the greater quantity of residual yeast DNA found in ‘Asprinio_B’ with respect to ‘Asprinio_A’, considering both vintages, and other factors, such as grapevine training systems.

The main relevance of this study is the characterization of ‘Asprinio’ wine produced with the ‘vite maritata’ training system. Indeed, although nowadays no information is reported in the literature, this peculiar method is currently under administration to be recognized as a Unesco World Heritage due to the spectacular ‘Alberata Aversana’.

In light of this, further work will be carried out in order to characterise the microorganisms’ population involved in wine fermentation, which probably changes depending on the different heights of the vine shoots, according to the training systems used. This is of interest as it highlights the need to verify the Asprinio vine training system used, not only to preserve it, but also because the flavour of ‘Asprinio’ wine could vary based on it.

## Figures and Tables

**Table 1:** Colourimetric parameters (WC, wine colour; WPPC, wine polymeric pigment content; WCP, wine colour pigment), total tannins content (TTC) and total flavonoids content (TFC) of wine samples. Values are expressed as mean ± SD (*n* = 3).

	‘Asprinio_A’		‘Asprinio_B’		‘Greco di Tufo’	
	2019	2020	2019	2020	2019	2020
WC (AU)	0.0303 ± 0.0010	0.0356 ± 0.0084	0.0352 ± 0.0018	0.0336 ± 0.0024	0.0128 ± 0.0002	0.0086 ± 0.0002
WPPC (AU)	0.0254 ± 0.0021	0.0284 ± 0.0059	0.0293 ± 0.0035	0.0278 ± 0.0033	0.0077 ± 0.0006	0.0102 ± 0.0013
WCP (AU)	0.0028 ± 0.0006	0.0028 ± 0.0003	0.0033 ± 0.0001	0.0023 ± 0.0003	0.0027 ± 0.0013	0.0037 ± 0.0020
Red (%)	16.81 ± 0.21	19.79 ± 0.68	19.07 ± 0.15	19.99 ± 0.21	14.05 ± 0.12	12.49 ± 0.45
Yellow (%)	80.16 ± 0.18	74.80 ± 1.89	77.30 ± 0.014	75.95 ± 0.87	83.62 ± 1.04	86.21 ± 1.26
Blue (%)	3.04 ± 0.02	5.41 ± 1.21	3.63 ± 0.012	4.08 ± 0.66	2.35 ± 0.91	1.29 ± 0.81
TTC (g/L)	0.356 ± 0.069	0.551 ± 0.376	0.688 ± 0.1032	0.728 ± 0.341	0.145 ± 0.030	0.145 ± 0.037
TFC (mg/L)	164.8 ± 4.84	226.6 ± 4.53	164.8 ± 4.65	226.6 ± 4.52	164.8 ± 4.75	230.0 ± 4.50

**Table 2:** Free amino acids composition in ‘Asprinio_A’, ‘Asprinio_B’ and ‘Greco di Tufo’, considering the 2019 and 2020 vintages. Values are means ± SD (*n* = 3) and expressed as mg per L of wine.

Free AA (mg/L)	‘Asprinio_A’		*p* Value	‘Asprinio_B’		*p* Value	‘Greco di Tufo’		*p* Value
	2019	2020		2019	2020		2019	2020	
Protein amino acids									
Asp	8.75 ± 1.14 a	8.04 ± 0.25 a	>0.05	2.84 ± 0.20 a	6.52 ± 0.07 b	<0.001	8.56 ± 1.18 a	8.47 ± 0.05 a	>0.05
Thr	3.30 ± 0.72 a	1.67 ± 0.01 a	>0.05	1.37 ± 0.21 a	0.65 ± 0.04 a	>0.05	3.75 ± 0.32 a	2.79 ± 0.04 a	>0.05
Ser	8.38 ± 0.33 a	1.71 ± 0.01 a	>0.05	7.43 ± 0.17 a	0.86 ± 0.06 b	<0.001	8.47 ± 0.27 a	2.54 ± 0.01 a	>0.05
Asn	6.03 ± 0.22 a	4.60 ± 0.14 a	>0.05	1.91 ± 0.19 a	2.21 ± 0.30 a	>0.05	14.18 ± 1.99 a	9.35 ± 0.25 a	>0.05
Glu	14.68 ± 0.60 a	7.34 ± 0.21 a	>0.05	10.27 ± 0.87 a	5.21 ± 0.23 b	<0.001	18.81 ± 1.54 a	14.32 ± 0.30 a	>0.05
Gln	11.67 ± 0.93 a	2.05 ± 0.14 b	<0.01	10.87 ± 0.49 a	1.79 ± 0.11 b	<0.001	11.17 ± 0.51 a	3.37 ± 0.06 b	<0.05
Gly	2.33 ± 0.13 a	1.28 ± 0.06 a	>0.05	0.90 ± 0.14 a	0.56 ± 0.03 a	>0.05	4.47 ± 0.38 a	3.24 ± 0.10 a	>0.05
Ala	10.17 ± 0.52 a	5.87 ± 0.04 a	>0.05	4.94 ± 0.16 a	2.07 ± 0.04 b	<0.001	15.37 ± 0.49 a	10.60 ± 0.27 a	>0.05
Val	2.68 ± 0.08 a	2.46 ± 0.11 a	>0.05	1.23 ± 0.19 a	1.03 ± 0.07 a	>0.05	4.17 ± 0.13 a	3.25 ± 0.16 a	>0.05
Met	1.90 ± 0.08 a	2.21 ± 0.02 a	>0.05	0.75 ± 0.08	n.d.		2.92 ± 0.12 a	2.61 ± 0.13 a	>0.05
Ile	3.54 ± 0.39 a	2.28 ± 0.09 a	>0.05	2.01 ± 0.24 a	1.20 ± 0.05 a	>0.05	5.08 ± 0.34 a	2.93 ± 0.05 a	>0.05
Leu	11.19 ± 0.46 a	5.62 ± 0.20 a	>0.05	4.84 ± 0.08 a	2.10 ± 0.08 b	<0.001	12.12 ± 0.57 a	8.42 ± 0.26 a	>0.05
Tyr	7.37 ± 0.68 a	2.55 ± 0.29 a	>0.05	2.52 ± 0.25 a	1.07 ± 0.08 a	>0.05	6.08 ± 0.65 a	3.84 ± 0.16 a	>0.05
Phe	8.88 ± 0.44 a	3.65 ± 0.02 a	>0.05	3.04 ± 0.21 a	1.32 ± 0.53 a	>0.05	8.33 ± 0.64 a	4.99 ± 0.22 a	>0.05
Lys	15.93 ± 0.87 a	6.42 ± 0.27 b	<0.01	4.98 ± 0.88 a	1.63 ± 0.17 b	<0.001	14.77 ± 1.96 a	7.30 ± 0.20 b	< 0.05
His	6.13 ± 0.89 a	1.50 ± 0.21 a	>0.05	1.65 ± 0.02 a	0.34 ± 0.09 a	>0.05	4.42 ± 0.64 a	1.57 ± 0.17 a	>0.05
Arg	13.76 ± 1.51 a	5.04 ± 0.21 b	<0.01	2.68 ± 0.85 a	1.35 ± 0.24 a	>0.05	11.08 ± 1.99 a	13.37 ± 0.44 a	>0.05
Non-protein amino acids									
Phser	7.84 ± 0.64 a	6.30 ± 0.02 a	>0.05	5.43 ± 0.64 a	4.42 ± 0.28 a	>0.05	7.44 ± 0.74 a	6.48 ± 0.03 a	>0.05
Taur	4.74 ± 0.33 a	2.60 ± 0.02 a	>0.05	3.41 ± 0.18 a	2.04 ± 0.08 a	>0.05	3.72 ± 0.18 a	2.94 ± 0.05 a	>0.05
β-ala	10.62 ± 0.53 a	4.60 ± 0.21 a	>0.05	4.35 ± 0.31 a	2.36 ± 0.10 b	<0.05	5.44 ± 0.66 a	3.77 ± 0.02 a	>0.05
Baiba	2.61 ± 0.21 a	0.72 ± 0.06 a	>0.05	0.83 ± 0.20	n.d.		2.04 ± 0.35 a	1.05 ± 0.03 a	>0.05
Homocys	6.06 ± 0.99 a	2.16 ± 0.07 a	>0.05	4.25 ± 0.64 a	0.80 ± 0.53 b	<0.001	5.71 ± 1.33 a	1.71 ± 0.07 a	>0.05
Gaba	1.73 ± 0.16 a	2.47 ± 0.02 a	>0.05	3.59 ± 0.19 a	1.59 ± 0.05 b	<0.05	10.27 ± 0.48 a	8.00 ± 0.29 a	>0.05
Ethan	8.59 ± 1.92 a	6.59 ± 0.07 a	>0.05	10.28 ± 0.31 a	8.46 ± 0.22 a	>0.05	8.40 ± 1.22 a	6.89 ± 0.22 a	>0.05
Orn	0.61 ± 0.14	n.d.		0.91 ± 0.10 a	0.19 ± 0.01 a	>0.05	1.56 ± 0.31 a	0.68 ± 0.08 a	>0.05
Sub-total	179.48	89.72		97.28	49.67		198.34	134.46	
Pro	179.83 ± 10.16 a	281.58 ± 12.80 b	<0.001	172.84 ± 3.00 a	225.33 ± 5.84 b	<0.001	152.88 ± 6.96 a	234.56 ± 17.87 b	<0.01
Total	359.31	371.30		270.12	275.00		351.22	369.02	

Three-letter code has been used; n.d., not detected. Values followed by different letters within a row are significantly different by the Bonferroni post-test.

## Data Availability

The data presented in this study are available on request from the corresponding author.

## Supplementary Materials

The following supporting information can be downloaded at: https://www.mdpi.com/article/10.3390/foods11152322/s1, Figure S1. Representative images of the different training systems for the ‘Asprinio’ grape variety. (a), traditional ‘vite maritata’ training system used for ‘Asprinio_A’; (b), guyot training system used for ‘Asprinio_B’; Table S1. Description of white wine samples analysed. Information given in this Table was get from labels on the bottle of wines; Table S2. Primers used in this study for quantitative PCR analysis (see Section 2.7.2); Table S3. Main metabolites detected in wines. ^1^H-NMR data are measured in ppm and coupling constants (J) in Hertz.
