# Supplementary material for: Biochemical Traits, 1H NMR Profile and Residual DNA Content of ‘Asprinio’, White Wine from Campania Region (Southern Italy)
Source: Foods. 2022 Aug 3;11(15):2322. doi: 10.3390/foods11152322 (PMC9368296; doi:10.3390/foods11152322)
Supplement: Supplementary file 1 [file foods-11-02322-s001.zip › foods-1792697-supplementary.pdf]

Biochemical traits, ^{1}H NMR profile and residual DNA content of 'Asprinio', white wine from Campania region (Southern Italy)

Nicola Landi, Monica Scognamiglio, Pasqualina Woodrow, Loredana F. Ciarmiello, Sara Ragucci, Angela Clemente, Hafiza Z. F. Hussain, Antonio Fiorentino and Antimo Di Maro *

Department of Environmental, Biological and Pharmaceutical Sciences and Technologies (DiSTABiF), University of Campania 'Luigi Vanvitelli', Via Vivaldi 43, 81100 Caserta, Italy

* Correspondence: antimo.dimaro@unicampania.it; Tel: +39-0823-274409

Figure S1. Representative images of the different training systems for ‘Asprinio’ grape variety. (a), traditional ‘*vite maritata*’ training system used for the ‘Asprinio_A’; (b), guyot training system used for the ‘Asprinio_B’.

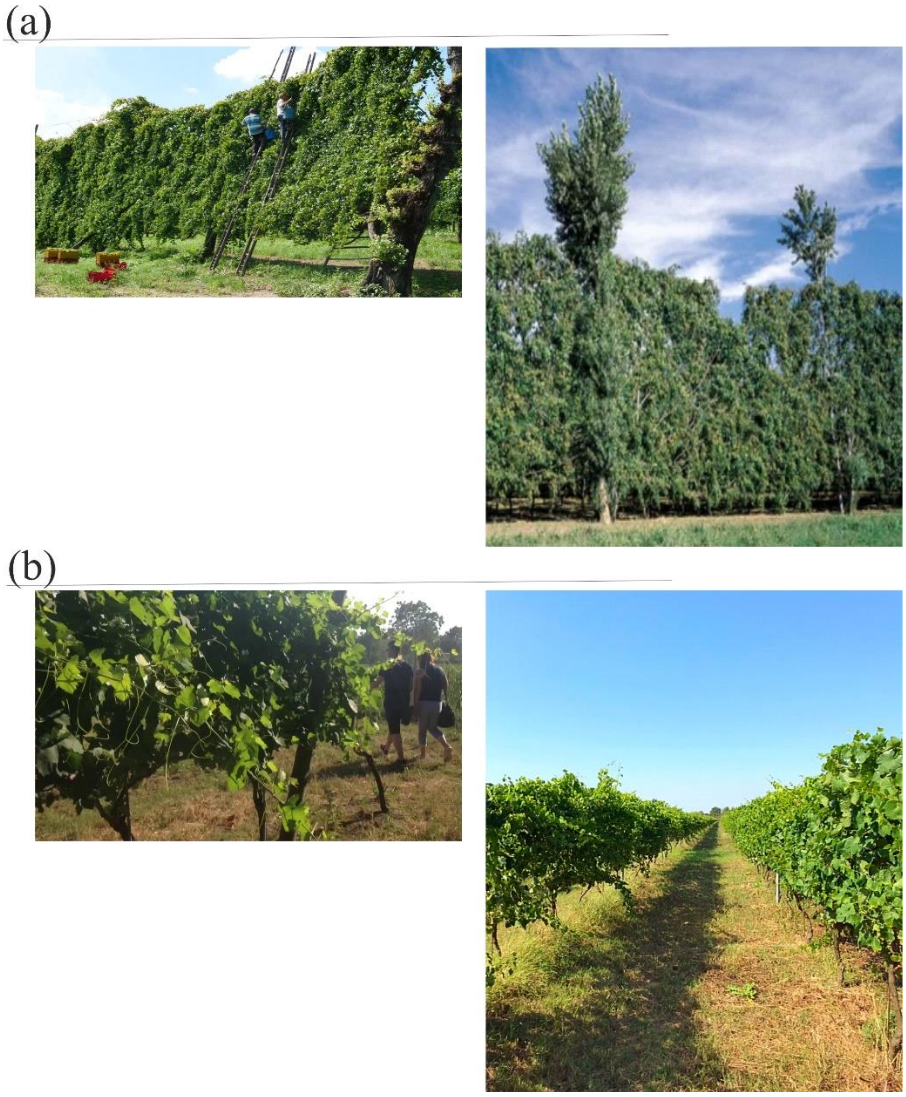

Table S1. Description of white wine samples analysed. Information given in this Table was get from labels on the bottle of wines.

		'Asprinio_A'	'Asprinio_B'	'Greco di Tufo'
Alcohol (%)	2019	12.0	12.0	12.5
	2020	12.0	11.5	12.5
pH *	2019	3.15±0.02	3.46±0.02	3.18±0.01
	2020	3.20±0.02	3.34±0.01	3.25±0.02

*, pH value was determined experimentally in our laboratory.

Table S2. Primers used in this study for quantitative PCR analysis (see paragraph 2.7.2).

Primer name	Sequences 5' > 3'
VvNCED2 F	ATGGCGACGGTATGGTTCA
VvNCED2 R	CGCTCCTGGACCAATCTCTG
ScRPS3 F	CCAACCAAGACCGAAGTTAT
ScRPS3 R	CCTGGAGCGTACTTGAATCT
ITS-S2 F	ATGCGATACTTGGTGTGAAT
ITS4 R	TCCTCCGCTTATTGATATGC

Table S3. Main metabolites detected in wines. ^{1}H-NMR data are measured in ppm and coupling constants (J) in Hertz.

**Metabolites**	**NMR**
Acetic Acid	2.08 (s)
Citric Acid	2.78 (d, *J*= 17.6); 2.93 (d, *J*= 17.6)
Malic Acid	2.76 (dd, *J*= 15.6, 9.3); 2.88 (dd, *J*= 15.6, 3.6); 4.51 (dd, *J*= 9.3, 3.6)
Succinic Acid	2.65 (s)
Tartaric Acid	4.55 (s)
Alanine	1.47 (d *J*= 7.2)
Phenylalanine	7.32 d (J = 7.5 Hz); 7.36–7.38 (m)
Proline	1.99 (m); 2.06 (m); 2.34 (m)
Tyrosine	6.93 (J = 7.7 Hz); 7.17 (J = 7.7 Hz),
Glucose	4.59 (β, d, *J*= 7.8); 5.19 (α, d, *J*= 3.8)
Sucrose	4.15 (ov); 5.30 (d, *J*= 3.6)
Caffeic acid	6.43 (d, J = 16.0); 6.84 (d, J = 8.0); 7.14 (dd, J = 8.0 and 2.0); 7.21 (d, J = 2.0); 7.68 (d, J = 16.0)
Trigonelline	9.13 (s); 8.82 (m); 8.08 (m)

Signal multiplicity indicated as: d=doublet, dd= doublet of doublets, m= multiplet, ov= overlapped, s= singlet
